# Neutralization of Zika virus by germline-like human monoclonal antibodies targeting cryptic epitopes on envelope domain III

**DOI:** 10.1038/emi.2017.79

**Published:** 2017-10-11

**Authors:** Yanling Wu, Shun Li, Lanying Du, Chunyu Wang, Peng Zou, Binbin Hong, Mengjiao Yuan, Xiaonan Ren, Wanbo Tai, Yu Kong, Chen Zhou, Lu Lu, Xiaohui Zhou, Shibo Jiang, Tianlei Ying

**Affiliations:** 1 Key Laboratory of Medical Molecular Virology of Ministries of Education and Health, School of Basic Medical Sciences, Fudan University, Shanghai 200032, China; 2 Shanghai Public Health Clinical Center, Fudan University, Shanghai 201508, China; 3 Lindsley F Kimball Research Institute, New York Blood Center, New York, NY 10065, USA; 4 Biomissile Corporation, Shanghai 201203, China

**Keywords:** cryptic epitope, human monoclonal antibodies, Zika virus

## Abstract

The Zika virus (ZIKV), a flavivirus transmitted by *Aedes* mosquitoes, has emerged as a global public health concern. Pre-existing cross-reactive antibodies against other flaviviruses could modulate immune responses to ZIKV infection by antibody-dependent enhancement, highlighting the importance of understanding the immunogenicity of the ZIKV envelope protein. In this study, we identified a panel of human monoclonal antibodies (mAbs) that target domain III (DIII) of the ZIKV envelope protein from a very large phage-display naive antibody library. These germline-like antibodies, sharing 98%–100% hoLogy with their corresponding germline IGHV genes, bound ZIKV DIII specifically with high affinities. One mAb, m301, broadly neutralized the currently circulating ZIKV strains and showed a synergistic effect with another mAb, m302, in neutralizing ZIKV *in vitro* and in a mouse model of ZIKV infection. Interestingly, epitope mapping and competitive binding studies suggest that m301 and m302 bind adjacent regions of the DIII C–C′ loop, which represents a recently identified cryptic epitope that is intermittently exposed in an uncharacterized virus conformation. This study extended our understanding of antigenic epitopes of ZIKV antibodies and has direct implications for the design of ZIKV vaccines.

## INTRODUCTION

The Zika virus (ZIKV) is a re-emerging mosquito-borne enveloped virus belonging to the genus Flavivirus, a genus that has recently received considerable attention owing to the rapidly increasing incidence of dengue (DENV), yellow fever, West Nile, and tick-borne encephalitis virus infections.^1^ Since its discovery in 1947, ZIKV has been described as a relatively mild pathogen that only causes sporadic infections in humans.^2, 3^ However, the geographic distribution and epidemic activity of ZIKV infection increased dramatically after the first large outbreak of the virus in 2007.^4^ The recent 2015–2016 outbreak in Latin America is the largest Zika outbreak in history, with over one million cases of infection across 67 countries; 53 of those countries are experiencing their first Zika outbreak since 2015.^5, 6^ Moreover, during this outbreak, ZIKV infection was linked to various developmental and neurological conditions, such as microcephaly and Guillain–Barré syndrome, raising serious concerns about its continued global spread.^7, 8^ No preventive or therapeutic products are approved or currently available; therefore, effective antivirals and vaccines are urgently needed to control and prevent ZIKV infections.

Understanding the human antibody response to ZIKV is central to the development of effective vaccines and serodiagnostics.^9, 10, 11, 12^ Three-dimensional cryo-electron microscopy structures of ZIKV reveal that it shares a high structural similarity to other flaviviruses with known structures.^13, 14^ The ZIKV envelope (E) glycoprotein, which was found to be a dimer on the surface of a mature virion, mediates viral entry, membrane fusion, and serves as the major target for neutralizing antibodies.^15^ Recent studies have revealed that critical targets of neutralizing antibody responses to flaviviruses are conformational or quaternary epitopes that require higher-order structures not presented on E monomers.^16, 17, 18^ Similar to other flaviviruses, the E protein of ZIKV comprises three domains: a central β-barrel domain (domain I, DI), an extended dimerization domain (domain II, DII), and an immunoglobulin-like domain (domain III, DIII). Previous studies on DENV-challenged mice showed that DIII-specific antibodies form a significant fraction of neutralizing antibodies and possess highly neutralizing activity.^19, 20^ Intriguingly, it was found that the neutralizing human antibodies mainly recognize quaternary structure-dependent epitopes presented on the intact virion in human immune sera;^21^ however, the majority of antibodies produced during a primary infection of individuals are non- or poorly neutralizing antibodies that are typically recognized by the fusion loop or *bc*-loop on DII of the E protein^22, 23^ and may cause antibody-dependent enhancement.^6, 24^ For instance, pre-existing DENV immunity was found to be highly cross-reactive to ZIKV and potently increase the subsequent infection of ZIKV.^6, 24^ These findings highlight the importance of directing antibodies specifically against ZIKV DIII to prevent and treat ZIKV infection.

A number of potent neutralizing monoclonal antibodies (mAbs) targeting ZIKV DIII have been recently described, but all were isolated from ZIKV-infected mice or individuals.^25, 26, 27, 28^ The current study sought to develop ZIKV DIII-specific fully human mAbs with novel properties and minimal divergence from their germline predecessors. To do this, we chose recombinant ZIKV envelope DIII as the antigen for panning a very large naïve IgM library (containing ~10^11^ antibodies) constructed from the blood of healthy adult donors. We identified a panel of antibodies that bound ZIKV DIII with high avidities ranging from low-nanoLar to subnanoLar and exhibited weak to moderate neutralizing potency. The most potent mAb, m301, broadly neutralized the American and Asian ZIKV strains circulating in the 2015–2016 outbreak. Combination of m301 with another mAb, m302, showed a synergistic effect in neutralizing ZIKV *in vitro* and in a mouse model of ZIKV infection. Interestingly, m301 and m302 bound adjacent regions of the DIII C–C′ loop, which represents a recently identified cryptic epitope according to the structure of the mature flavivirus virion. Though cryptic, this epitope is speculated to be intermittently exposed in an uncharacterized virus conformation. Further immunogenetic analysis suggests that m301 and m302 are germline-like antibodies with ~98% identity to the germline IGHV3–30 gene and that the fast and effective elicitation of such antibodies by immunization is promising. These results may be relevant for the understanding of antigenic epitopes of ZIKV antibodies, and the design of effective ZIKV vaccines and serodiagnostics.

## MATERIALS AND METHODS

### Viruses

Asian ZIKV strain SZ01, originally isolated from the blood of a Chinese patient who had traveled to Samoa in 2016, was obtained as a gift from Prof. Cheng-Feng Qin, Beijing Institute of Microbiology and Epidemiology. ZIKV strains R103451, PRVABC59, H/PAN/2015/CDC-259359 (PAN2015), and FLR were obtained from BEI Resources, NIAID, NIH (Bethesda, MD, USA). ZIKV strain R103451 was originally isolated on 6 January 2016 from the placenta of a human who had traveled to Honduras in 2015. PRVABC59 was isolated from the blood of a human in Puerto Rico in December 2015. PAN2015 was isolated from a serum specimen collected from a human in Panama on 18 December 2015. Finally, FLR was isolated from the blood of a human in Barranquilla, Colombia, in December 2015. Virus stocks were prepared by inoculation onto a confluent monolayer of Vero E6 cells or C6/36 mosquito cells and titrated by plaque-forming units (PFU) on Vero E6 cells.

### Preparation of ZIKV envelope DIII protein

The gene encoding ectodomain residues 303–404 of ZIKV Envelope protein DIII (ZIKV strain MR766) was synthesized by GENEWIZ and then cloned into the pSecTag B vector with AviTag. After sequences of the expression vectors were confirmed, the recombinant proteins were expressed transiently in 293 FreeStyle cultures. Recombinant ZIKV DIII protein was purified from the conditioned culture media and dialyzed into phosphate-buffered saline (PBS). The protein was biotinylated by the BirA biotin-protein ligase in PBS for 30 min on ice, which adds biotin covalently to AviTag in a highly specific manner, according to the manufacturer’s instructions (Pierce, Waltham, MA, USA).

### Bio-panning

The large phage-display naive human antigen-binding fragment (Fab) library was constructed using peripheral blood mononuclear cell complementary DNA from 40 healthy volunteers as the template for cloning the expressed antibody gene repertoire. Panning protocols were essentially carried out as previously described.^29^ Briefly, one aliquot of each of the frozen library phage stocks was precipitated with 5% polyethylene glycol (PEG) 8000-NaCl (20% PEG8000, 2.5 M NaCl) and resuspended in PBS. The recombinant biotinylated ZIKV DIII-hFc fusion protein was used for panning of the libraries. For screening, 5 μg of biotinylated antigen was utilized in round 1, 4 μg was utilized in round 2, 2 μg was utilized in round 3, and 1 μg was utilized in round 4, followed by immobilization on streptavidin-coated magnetic beads (Invitrogen, Waltham, MA, USA). Approximately 1 × 10^12^ phage particles were used for each bio-panning. Phages from the library were preblocked in 3% milk powder (w/v) in PBS (MPBS), incubated with the biotinylated antigen in 1% MPBS for 30 min, and then incubated with streptavidin-coated magnetic beads for 1.5 h. After washing with PBST (PBS buffer supplemented with 0.05% Tween 20), bound phages were used to infect mid-log phase *Escherichia coli* TG1 bacteria at 37 °C for 1 h. Then, the TG1 bacteria were grown in 2 × YT medium containing 100 μg/mL of ampicillin and 2% (w/v) glucose at 37 °C and 250 r/min. After 2 h, the cells were infected with 1 × 10^12^ VCSM13 helper phages for 45 min at room temperature. The infected cells were harvested and resuspended into 2 × YT medium supplemented with 100 μg/mL of ampicillin and 100 μg/mL of kanamycin. Cells were then incubated overnight at 30 °C and 220 r/min. The phages were precipitated from culture supernatant with PEG-8000-NaCl and resuspended in sterile PBS until the next panning. The enrichment for antigen-specific phages after each round of panning was assessed by polyclonal phage enzyme-linked immunosorbent assay (ELISA). Positive clones expressing Fab were identified from the third and fourth rounds of panning using monoclonal phage ELISA. The identified clones were analyzed and classified into different families based on their amino-acid sequence diversity in the complementarity-determining region (CDR) 3 region of the V_H_ or V_L_ gene.

### Enzyme-linked immunosorbent assay

Costar half-area high-binding assay plates (Corning, Corelle, NY, USA, #3690) were coated with purified DIII-hFc at 100 ng/well in PBS overnight at 4 °C and blocked with 3% milk powder (w/v) in PBS buffer at 37 °C. For phage ELISAs, phage from each round of panning (polyclonal phage ELISA), or clones randomly picked from the third and fourth rounds of panning-infected TG1 cells (monoclonal phage ELISA), were incubated with immobilized antigen. Bound phages were detected with anti-M13-horseradish peroxidase (HRP) polyclonal antibody (Pharmacia, Piscataway, NJ, USA). For the soluble Fab binding assay, serially diluted antibodies were added and incubated for 1.5 h at 37 °C. HRP-conjugated mouse anti-Flag (Sigma-Aldrich, St Louis, MO, USA) was used for detection. For the binding capacity of m301 with wild-type or mutant DIII, the plate was coated with m301 Fab. For generation of a panel of DIII mutants, the wild-type DIII encoding gene was cloned into pMAL-4 vectors, which encode maltose-binding protein (MBP), resulting in the expression of an MBP fusion protein. Then, mutants of DIII were generated using a site-directed mutagenesis kit (Yeasen, Inc., Shanghai, China) and expressed in *E. coli*. Wild-type and mutant DIII (50 μg/mL) were incubated, and HRP-conjugated anti-MBP (NEB, Inc., Ipswich, MA, USA) was used for detection. Enzyme activity was measured with the subsequent addition of substrate ABTS, and signal reading was carried out at 405 nm.

### Protein expression and purification

Fab expression was performed in *E. coli* HB2151 bacterial culture according to a previously published protocol and then purified on a Ni-NTA column.^30^ Recombinant Fabs carried Flag and His_6_ tags on their C-termini. For conversion and preparation of IgG1s, the heavy and light chains of Fabs were amplified and recloned into the PTT-IgG1 vector. The proteins were expressed transiently in Expi293 cultures and purified with a protein G column. Proteins were dialyzed into PBS. Purity was estimated as >95% by SDS–polyacrylamide gel electrophoresis, and protein concentration was measured spectrophotometrically.

### Western blot analysis

ZIKV DIII-hFc protein (5 μg/lane) was separated by 4%–12% SDS–polyacrylamide gel electrophoresis and transferred onto a nitrocellulose membrane (Millipore, Billerica, MA, USA). After washing with TBS-T buffer (20 mM Tris-HCl, 150 mM NaCl, 0.05% Tween-20), the membrane was blocked with 5% skim milk at room temperature for 1 h and then incubated with the Fabs overnight at 4 °C. After washing with TBS-T, the membrane was incubated with HRP-conjugated mouse anti-Flag (Sigma-Aldrich) for 2 h at room temperature. The membrane was washed with TBS-T and exposed using a chemiluminescent detection kit (CWBio, Inc., Beijing, China).

### Neutralization assay

Neutralizing activity of an IgG1 mAb was measured using a plaque reduction neutralization test assay with Vero E6 cells as previously described.^31, 32, 33^ As the positive control, the mouse sera were produced by immunizing mice with the 293T-expressed recombinant ZIKV E glycoprotein. Briefly, mAb was diluted in duplicate in a six-well plate in MEM supplemented with 1% fetal calf serum. An equal volume of ZIKV (approximately 200 PFU) was added and incubated for 1 h at 37 °C. Following incubation, the mixture was then layered onto Vero E6 cells in a six-well plate and incubated at 37 °C for 1.5 h. The infected cells were washed with PBS, overlaid with carboxyl-methyl cellulose, and incubated at 37 °C for 5 days. Cells were fixed and stained with 2 mL of 1% (w/v) crystal violet-formaldehyde solution, and the plaques were counted. Percent neutralization was calculated based on the percent reduction of plaques in the presence and absence of mAb or ZIKV E serum control.

### Biolayer interferometry binding assays

The binding kinetics of mAbs (m301, m302, m303, m304) with DIII of ZIKV E protein or an irrelevant protein (influenza HA, H3N2) was analyzed by biolayer interferometry using an Octet-Red96 device (Pall ForteBio, Menlo Park, CA, USA). Purified ZIKV-DIII-hFc or H3N2-HA at 30 μg/mL buffered in sodium acetate (pH 5.0) was immobilized onto activated AR2G biosensors and incubated with threefold serial dilutions of antibodies in running buffer. The experiments included the following steps at 37 °C: (1) equilibration (60 s); (2) activation of AR2G by 1-ethyl-3-(3-dimethylaminopropyl)carbodiimide hydrochloride/N-hydroxysuccinimide (300 s); (3) immobilization of protein onto sensors (300 s); (4) quenching with ethanolamine (300 s); (5) baseline (120 s); (6) association of antibodies for measurement of *K*_on_ (300 s); and (7) dissociation of antibodies for measurement of *K*_off_ (1500 s). Fitting curves were constructed using ForteBio Data Analysis software (Pall ForteBio).

For the competition assay, soluble ZIKV-DIII-hFc was bound to AR2G sensors. The association of each mAb at 100 nM was measured for 300 s at 37 °C, and then secondary antibodies (100 nM) were added in the presence of the first mAb.

### Molecular modeling and docking

The amino-acid sequences of the mAbs’ heavy- and light-chain variable domains (V_H_ and V_L_) were independently aligned with the corresponding primary sequences of all immunoglobulins deposited in the Protein Data Bank using the SWISS Model website. The best match for m301 was the human germline 5I1L^34^ with 97.35% identity of amino-acid residues. The most homology m302 shared was 92.52% identity with 5I1J. The crystal structures of DIII of the ZIKV E protein (PDB identifier 5kvg) originated from Zhao *et al.* as described. The initial models for the variable domains were generated based on the crystallization structure of the chosen templates. The Discover Studio module with a consistent valence force field was used to add water molecules and perform an energy minimization. Docking of the m301 and m302 models with DIII of the ZIKV E protein was carried out using Z-DOCK, and the top 10 optimal complexes were considered. All structural representations were colored and rendered using the PyMOL Molecular Graphics System (DeLano Scientific, San Carlos, CA, USA).

### Mouse experiments

C57BL/6 background mice deficient in alpha/beta interferon (IFN-α/β) and IFN-γ receptors (AG6 mice) were purchased from B&K Universal Group Limited (Shanghai, China) and housed under specific pathogen-free conditions at the animal facilities of the Shanghai Public Health Clinical Center, Fudan University (Shanghai, China). The mice were transferred to the Animal Biosafety Level 2 Laboratory (Shanghai, China) before infection. For antibody therapy against ZIKV infection, groups of mixed-sex 4- to 8-week-old mice were used for all experiments. All mice were intraperitoneally (i.p.) injected with 10^5^ PFU of ZIKV in a volume of 100 μL. At 12 h post infection, mice were passively transferred a single dose of 500 μg antibody m301, a cocktail of m301 and m302 (250 μg for each antibody), or PBS as the control via i.p. injection. For antibody protection from ZIKV infection, all mice were inoculated with a cocktail of antibodies (250 μg of m301 and m302) by i.p. injection. After 4 h, these mice were infected with 10^3^ PFU of ZIKV. Survival, weight loss, and disease signs were monitored daily.

### Data analysis

IMGT/High V-QUEST (version 1.5.1) was used for sequence annotation and assignment of the V(D)J genes. The results from IMGT/High V-QUEST analysis were imported into the PostgreSQL database, and Structured Query Language was used to retrieve the data for analysis. Other experimental data were analyzed with GraphPad Prism 6 software. Kaplan–Meier survival curves were analyzed by the log-rank test, and weight losses were compared using analysis of variance with a multiple-comparisons test. A *P* value of <0.05 indicated statistically significant differences.

### Ethics statement

All experimental protocols were approved by the institutional committee of Fudan University. All methods were carried out in accordance with relevant guidelines and regulations. Informed consent was obtained from all subjects.

## RESULTS

### Isolation and characterization of ZIKV DIII-specific mAbs

**Figure 1:**
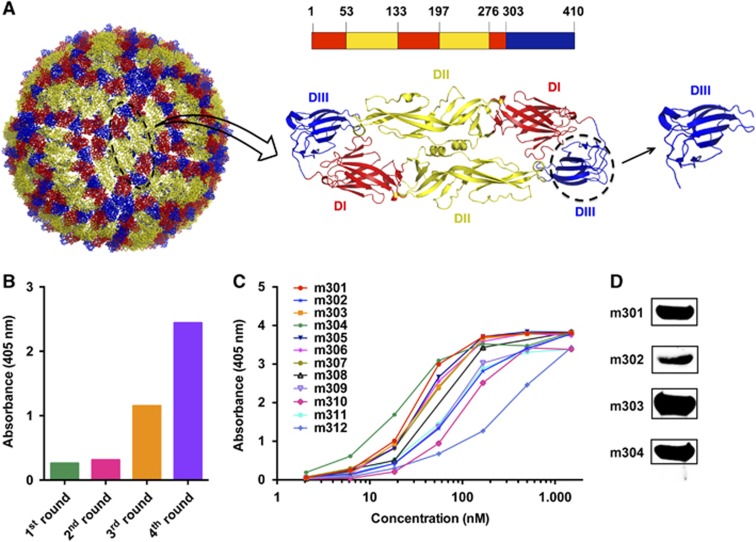
Bio-panning of anti-ZIKV DIII protein Fabs from the full-human Fab library. (**A**) The individual E protein (black oval), which consists of DI, DII and DIII, is indicated. DI, DII and DIII are shown in red, yellow, and blue, respectively. (**B**) Polyclonal phage ELISA showing the phage enrichment of four rounds (Rounds 1–4) by panning. (**C**) Binding of Fabs m301–m312 to ZIKV DIII protein according to ELISA. (**D**) Western blot analysis of binding activity for Fabs m301–m304. Domain, D; enzyme-linked immunosorbent assay, ELISA; Zika virus, ZIKV.

We previously prepared a large phage-display antibody Fab library using peripheral blood B cells of non-immunized healthy donors and identified panels of mAbs against viral and cancer-related targets.^29, 35, 36, 37^ In this study, we set out to isolate DIII-specific neutralizing mAbs against ZIKV using a similar methodology. ZIKV DIII was produced and biotinylated at a specific site for use as a target antigen during bio-panning (Figure 1A). Antibody selection from the naive human Fab library was performed as previously described.^29^ After four rounds of panning, potent enrichment was achieved, as indicated by a polyclonal phage ELISA (Figure 1B), and a panel of 12 DIII-specific Fabs with various binding affinities was identified using a soluble expression-based monoclonal ELISA (Figure 1C). Among these antibodies, four unique high-binding clones (m301, m302, m303, and m304) were selected for further characterization and converted into a full-length human IgG1 format. All antibodies were reactive to recombinant DIII or E glycoprotein in Western blot analyses, suggesting that m301–m304 mAbs might recognize linear epitopes on ZIKV DIII (Figure 1D).

**Figure 2:**
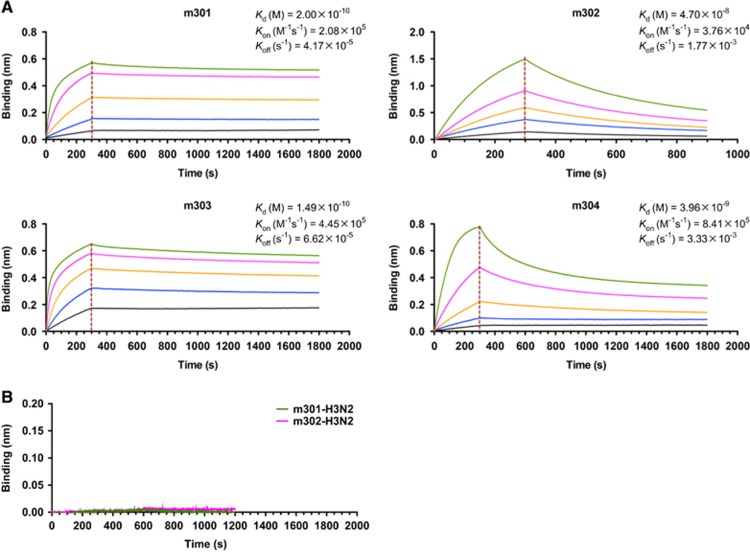
Binding kinetics of IgGs m301–m304 to ZIKV-DIII (**A**) or control antigen (**B**), as measured by BLI using OctetRED96. Purified ZIKV-DIII-hFc or control antigen (influenza H3N2 HA) was immobilized on activated AR2G biosensors. The analytes consisted of serial dilutions of IgGs between 100 and 1.2 nM. Binding kinetics were evaluated using a 1:1 Langmuir binding model by Fortebio Data Analysis 7.0 software. Biolayer interferometry, BLI; domain, D; immunoglobulin G, IgG; Zika virus, ZIKV.

We next measured the binding kinetics of these mAbs to the recombinant DIII protein using biolayer interferometry by Octet-RED (Pall ForteBio). As shown in Figure 2A, all four antibodies exhibited high DIII-binding avidities. The equilibrium dissociation constant (*K*_D_) of m301 for DIII was 0.2 nM with an on-rate (*k*_on_) of 2 × 10^5^ per M per s and off-rate (*k*_off_) of 4 × 10^−5^ per s. The m303 mAb displayed a binding pattern similar to that of m301 with a *K*_D_ of 0.15 nM. The m302 and m304 mAbs had much faster off-rates (*k*_off_ 2 × 10^−3^ and 3 × 10^−3^ per s, respectively), and m302 had the slowest on-rate among the four mAbs (*k*_on_ 4 × 10^4^ per M per s). Consequently, m302 had a measured *K*_D_ of 47 nM, and m304 had a *K*_D_ of 4 nM. The m301 and m302 mAbs showed no binding to influenza HA protein (Figure 2B), which suggests that the m301 and m302 binding could be specific.

### Sequence analysis

**Figure 3:**
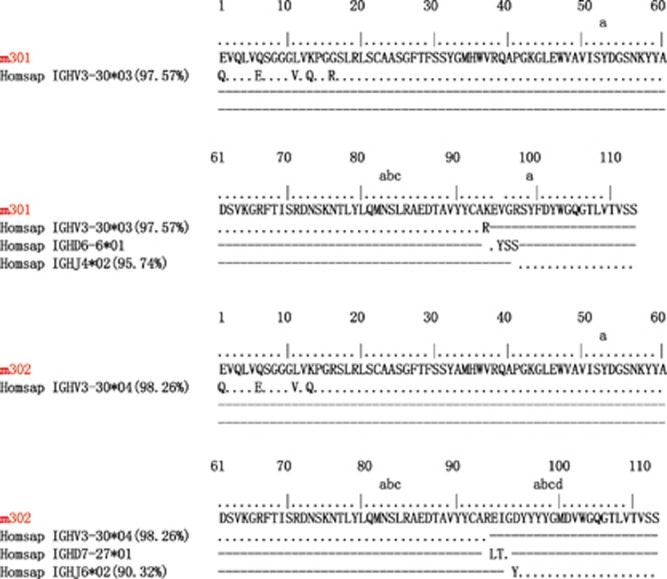
Immunogenetic analysis of the heavy- and light-chain variable regions of m301 and m302 using the IMGT tool.

Genetically, each of the four mAbs had unique V_H_ and V_L_ sequences. Immunogenetic analysis of their sequences was performed using the IMGT tool to determine the closest VH and VL germline genes (Figure 3, Table 1 and Supplementary Figure S1). Analyses of germline gene usage indicated that they originated from different B-cell lineages. Furthermore, their nucleotide sequences displayed 98%–100% IGHV identity to the germline (Table 1). The few somatic mutations were located in the N-terminus of the antibody heavy chain. These were positioned far from the CDR regions and did not appear to affect antigen binding (Figure 3 and Supplementary Figure S1). Therefore, all four antibodies were germline-like mAbs, which, in general, exhibit lower immunogenicity and better druggability properties compared to somatically hypermutated antibodies.^38^

While the patterns of VDJ gene segment usage were diverse among the four antibodies, three of them shared the same IGHV3–30 germline gene with slight differences between allelic variants (m301 and m304, IGHV3-30*03; m302, IGHV3-30*04). Interestingly, the germline V-gene IGHV3-30 was frequently used by a number of human neutralizing mAbs isolated from individuals infected with flaviviruses, including West Nile virus,^39^ DENV,^40^ and ZIKV.^41^ To further investigate the IGHV3-30 recombination frequency with specific IGHD and IGHJ genes among other germlines, we analyzed in detail the deep sequencing data of our antibodyome studies on naive IgM repertoires of 33 healthy adult donors and neonatal IgM repertoires of 10 newborn babies (unpublished). As expected, we observed a high IGHV3-30 recombination frequency with various IGHD and IGHJ genes. Notably, 32 unique VH clones were successfully identified as sharing a VDJ recombination pattern similar to that of m301 (IGHV3-30, IGHD6, IGHJ4) (Supplementary Figure S2). Thirty-three unique clones related to m302 (IGHV3-30, IGHD7, IGHJ6) and 16 unique clones related to m304 (IGHV3-30, IGHD3, IGHJ3) were also identified (Supplementary Figures S3 and S4). In contrast, only seven unique clones were related to m303, the antibody that utilized the IGHV3-64D germline gene (Supplementary Figure S5). Taken together, these results suggest that the elicitation of these germline-like antibodies, especially the IGHV3-30 lineage m301- and m302-like antibodies, could be relatively quick and effective by immunization of ZIKV DIII-based immunogens.

### Neutralizing activity against live ZIKV infection *in vitro*

**Figure 4:**
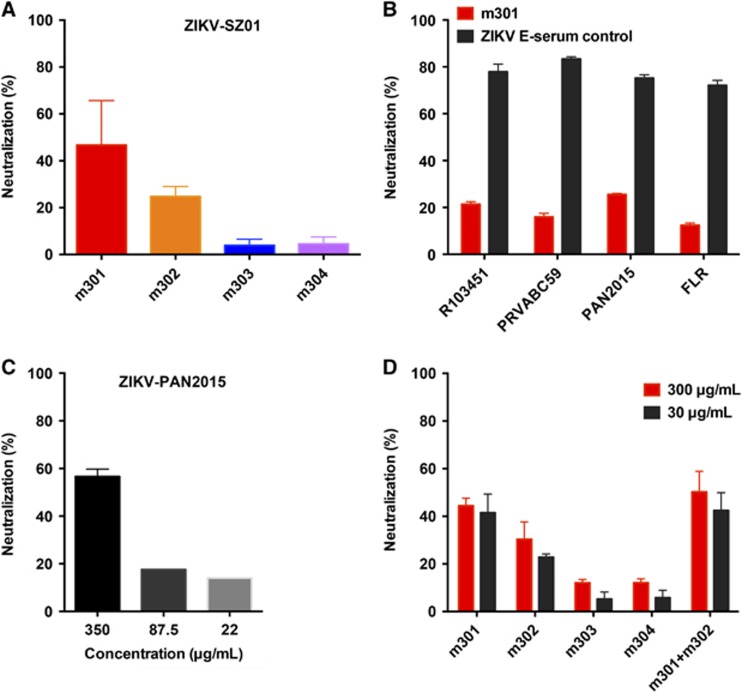
Neutralization activity of anti-ZIKV antibodies (m301–m304) to different ZIKV strains by plaque reduction assays. (**A**) The ZIKV Asian lineage SZ01 was incubated with 300 μg/mL m301–m304 antibodies. (**B**) Four kinds of ZIKV strains, including PAN2015, FLR, R103451, and PRVABC59, were incubated with 175 μg/mL m301. ZIKV E serum (1:50) served as a positive control. (**C**) PAN2015 was mixed with four-fold serial dilutions of m301 for 1.5 h at 37 °C prior to infection of Vero E6 cells. Subsequently, neutralization activity was evaluated by plaque reduction assays in duplicate. (**D**) Neutralization activity of mAbs alone or in a cocktail of m301 and m302 to ZIKV SZ01 strain. Data are represented as the mean±s.e.m. The figure represents three independent experiments performed in duplicate. Monoclonal antibodies, mAbs; Zika virus, ZIKV.

To examine the neutralizing capability of the four mAbs against ZIKV infection, we first evaluated the inhibitory activity of m301–304 using plaque reduction neutralization test against a recently isolated Asian lineage ZIKV, ZIKV SZ01.^31^ As shown in Figure 4A, m301 exhibited the highest neutralizing activity among the four mAbs. In comparison, m302 had modest neutralization activity, while m303 and m304 did not show any evident neutralization against ZIKV SZ01. To determine the breadth of neutralization of m301, we also tested its neutralizing activity against a panel of representative circulating ZIKV strains in addition to Asian lineage SZ01, including R103451 (Honduras strain), PRVABC59 (Puerto Rico strain), H/PAN/2015 (Panama strain), and FLR (Colombia strain). We found that the DIII of all these circulating strains is highly conserved, with only one mutation from the PRVABC59 strain (Supplementary Figure S6). Indeed, m301 was able to neutralize all these strains with similar potency, suggesting that m301 is a broadly neutralizing anti-ZIKV antibody (Figure 4B). The m301 mAb inhibited ZIKV infection in a dose-dependent manner with 2 μM resulting in ~57% neutralization by plaque reduction neutralization test, which was less effective than the control sera of mice immunized with recombinant ZIKV E glycoprotein (Figures 4B and 4C). Interestingly, neutralization of SZ01 does not differ with decreasing m301 concentrations (Figure 4D), suggesting that the neutralizing activity of m301 could vary from isolate to isolate.

### Competitive binding study

**Figure 5:**
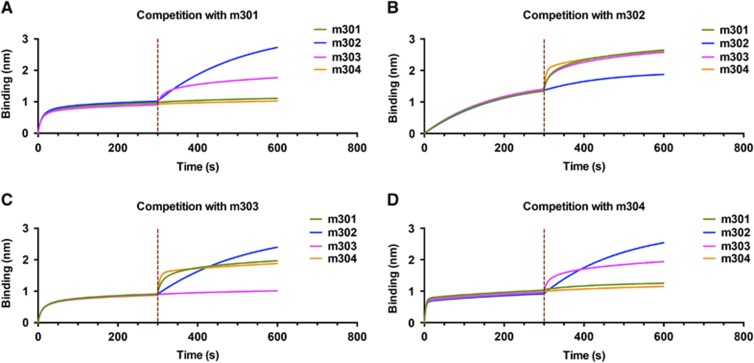
A competition assay was performed among different IgGs to DIII. Immobilized ZIKV-DIII-hFc was first saturated with 100 nM of m301 (**A**), m302 (**B**), m303 (**C**) or m304 (**D**). The capacity of the second IgG binding to the antigen was monitored by measuring further shifts after injecting the second IgG (100 nM) in the presence of the first IgG (100 nM). The red dotted vertical line represents the second IgG loading. domain, D; immunoglobulin G, IgG; Zika virus, ZIKV.

The observed difference among the four antibodies in neutralizing activity against live ZIKV could have resulted from the difference in binding avidities and recognized epitopes. To test this hypothesis, we performed binding competition experiments using biolayer interferometry. The biosensors labeled with ZIKV DIII were saturated with a mAb analyte in solution, followed by the addition of a second mAb in the presence of the first antibody. As shown in Figures 5A–5D, m301 competed with m304 for binding to DIII, but not with mAbs m302 and m303. Neither m302 nor m303 showed any competition with the other three mAbs. Furthermore, m302 exhibited a much slower binding phase compared to the other three mAbs, which is consistent with its 10-fold lower *k*_on_ value, as observed in the DIII binding assay (Figure 2). These data reveal three distinct epitopes on DIII. Although m301 and m304 may recognize overlapping epitopes, the much lower neutralizing activity of m304 could be attributed to its 20-fold lower DIII binding avidity. Taken together, these results suggest that m301 and m302 bind different epitopes on DIII and thus may have a synergistic effect in inhibiting ZIKV infection. Indeed, an enhancement in the neutralization of an antibody cocktail (m301 in combination with m302) was detected compared with that of either m301 or m302 alone at the same concentration, indicating a moderate synergistic effect of m301 and m302 on ZIKV neutralization (Figure 4D).

### Epitope mapping

**Figure 6:**
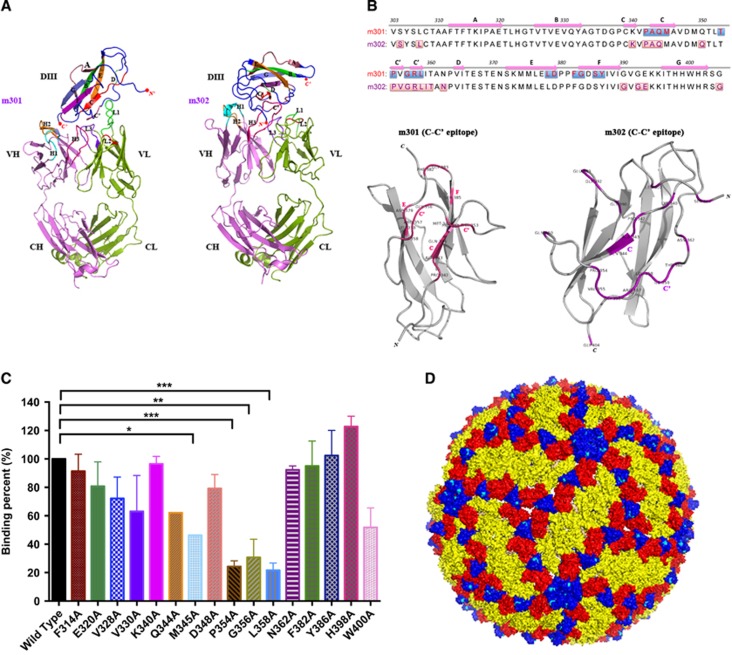
The structural basis and epitopes of anti-ZIKV Fabs m301 and m302 on ZIKV DIII. (**A**) Docking diagrams of m301 Fab (left) and m302 Fab (right) onto ZIKV DIII (PDB identifier 5 kvg) complexes with antibody fragments are shown. The heavy chain of Fab is in red, and the light chain is in green. DIII is colored dark blue with contact segments labeled. (**B**) Sequence definition of binding epitopes on ZIKV-specific DIII. DIII residues are colored if they make van der Waals contact within 3 Å distance, and the total number of contacts for each epitope residue are shown below the DIII sequences. (**C**) The binding capacity of m301 with wild-type and a panel of mutant DIII determined by ELISA. Data are represented as the mean±s.e.m. The figure represents three independent experiments performed in duplicate. (**D**) The structure of the mature ZIKV E protein (PDB 4ire) is shown. The C–C′ loop epitope residues are colored in cyan in each symmetry group. Domain, D; enzyme-linked immunosorbent assay, ELISA; antigen-binding fragment, Fab; Zika virus, ZIKV.

We previously developed a molecular docking-based strategy for computational prediction of neutralizing antibody epitopes on the receptor-binding domain of the MERS-CoV S glycoprotein.^29^ In a follow-up study, we determined the high-resolution complex structure of the antibody and MERS-CoV receptor-binding domain by X-ray crystallography and found that the previous docking predictions closely matched the crystallographic geometry, with most epitopes and critical interactions successfully predicted.^35^ Thus, in this study, we used a similar approach to map the binding epitopes of neutralizing mAbs m301 and m302 on ZIKV DIII. The structure of m301 was generated by homology modeling based on the crystal structure of a human germline antibody sharing 97% amino-acid sequence identity with m301, while m302 was built based on another germline antibody with 93% sequence identity.^34^ The structures were energy-minimized in a water box and then docked onto the crystal structure of the ZIKV E protein DIII.^25^ Interestingly, we found that both m301 and m302 engaged the ‘C–C′ loop’ encompassed by C- and C′-strands and their connecting loop, but in different directions and spatial locations (Figure 6A). As shown in Figure 6B, the epitopes of m301 and m302 on DIII were different, but mostly concentrated on the C–C′ loop (9 and 12 residues, respectively). This result explains the competitive binding between m301 and m302 on DIII, but relatively moderate synergistic effect when used in combination for ZIKV neutralization. Analysis of antibody contact residues indicates that m301 binding is dominated by light-chain CDR usage, whereas m302 primarily used heavy-chain CDRs engaging DIII.

To further localize the m301 epitopes, a panel of DIII alanine scanning mutants was generated. The binding of m301 to these mutants was measured by ELISA. Substitutions of four residues in the C–C′ loop could significantly reduce the binding activity of m301 to DIII, including M345, P354, G356, and L358 (Figure 6C), indicating that these four residues are involved in the epitopic composition.

Notably, the C–C′ loop epitope recognized by m301 and m302 represents a cryptic epitope that should not be exposed on the virion, according to existing flavivirus cryo-electron microscopy structures.^42, 43^ Indeed, we docked the DIII epitopes of m301 and m302 onto the cryo-electron microscopy structures of the mature ZIKV virion and found that the epitopes were buried inside and not accessible on the surface (Figure 6D). Interestingly, the binding pattern of m301 on ZIKV DIII remarkably resembled that of DENV neutralizing antibody E111 on DENV DIII, which was isolated from mouse serum after immunization with a strain of DENV-1 virus.^42, 44^ Similarly, the C–C′ loop epitope recognized by E111 was completely inaccessible in all DENV virion models. Therefore, it was hypothesized that the cryptic C–C′ loop epitope could be intermittently exposed in an uncharacterized virus conformation via a process named ‘viral breathing’. Through this process, antibodies targeting completely inaccessible epitopes on the mature virion can still neutralize flavivirus infection.^43^ Recently, mouse mAbs recognizing C–C′ loop epitopes were also identified after immunization with ZIKV.^25^ Therefore, it is reasonable to speculate that m301 and m302 target an uncharacterized virion structure and moderately neutralize ZIKV via viral breathing.

### *In vivo* protection study

Adult wild-type mice are not naturally susceptible to experimental infection with ZIKV.^45^ Recently, we developed a lethal mouse model of ZIKV infection using C57BL/6 mice deficient in IFN-α/β and γ receptors, named AG6 mice, which were successfully used in the establishment of a DENV infection mouse model.^46^ In this study, we used this model to assess whether neutralizing mAbs could protect against ZIKV infection *in vivo*.

**Figure 7:**
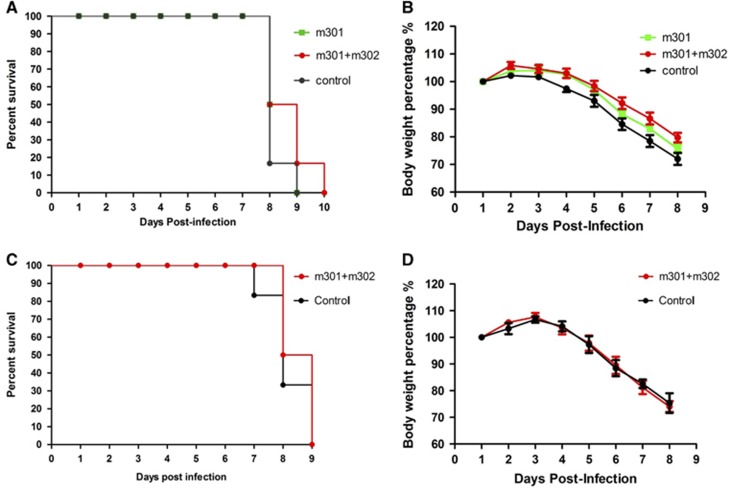
*In vivo* protection and therapeutic activity of anti-ZIKV mAbs against ZIKV infection. Four- to eight-week-old AG6 mice were inoculated with 10^5^ PFU of ZIKV by i.p. route. At 12 h post ZIKV infection or 4 h before 10^3^ PFU ZIKV infection, mice were administered m301 or a cocktail of m301 and m302 via i.p. injection. (**A**, **C**) Mortality of AG6 mice was monitored after infection. (**B**, **D**) Mice were weighed daily, and weights are expressed as the percentage of body weight prior to infection. Abbreviations: intraperitoneal, i.p.; monoclonal antibodies, mAbs; plaque-forming units, PFU; Zika virus, ZIKV.

To determine the therapeutic potential of the neutralizing mAbs, groups of mice (*N*=6 per group) were i.p. challenged with 10^5^ PFU of Asian ZIKV strain SZ01. Twelve hours later, they were treated (i.p.) with a single dose of either 0.5 mg m301 or a cocktail of m301 and m302 (each 0.25 mg) or PBS (control). This was followed by daily monitoring for well-being (weight loss and other clinical manifestations) and mortality. In the first 6 days, mice in the antibody groups were significantly more active and healthier than mice in the control group. At day 7, all mice in the control group suffered from complete loss of mobility, while only two mice in the m301 group and one in the cocktail antibody group appeared to have signs of limb paralysis. All others appeared healthy. However, all mice died within 10 days in spite of the considerable difference in clinical manifestations between the antibody groups and control group (Figures 7A and 7B). At day 8, five of six mice from the control group (16.6% survival) died, and three of six mice from the m301 group (50% survival) or cocktail antibody group (50% survival) died. At day 9, all mice from the control group and m301 group had died, while only one mouse from the cocktail antibody group (16.6% survival) survived. The last mouse died at day 10. Evidently, the neutralizing mAbs showed some therapeutic efficacy but were unable to eradicate the virus entirely, possibly because of their moderate neutralizing activities and/or extremely high viral loads in the AG6 animal model.

A recent study revealed that IFN-deficient mice are highly susceptible to lethal ZIKV infection.^47^ Therefore, to evaluate prophylactic efficacy, AG6 mice were inoculated with a cocktail of antibodies (0.25 mg m301 and 0.25 mg m302) and then challenged (i.p.) 4 h later with 10^3^ PFU of ZIKV strain SZ01, instead of 10^5^ PFU as used in the therapeutic study. Similar to the previous challenge, 10^3^ PFU of ZIKV also resulted in 100% mortality within 9 days (Figures 7C and 7D). One mouse from the control group died at day 7, and three more died at day 8 (33.3% survival). In comparison, three of six mice from the cocktail antibody group died at day 8 (50% survival). The remaining three mice did not show signs of limb paralysis, but they did exhibit reduced mobility, albeit to a lesser extent than the remaining two mice in the control group. The absence of significantly prolonged survival in the treatment groups could be attributed to the high sensitivity of the IFN-deficient mice to ZIKV infection. Residual virus load could also cause lethal infection in mice in the absence of complete neutralization of viruses by the anti-ZIKV mAbs. This interpretation is supported by a recent finding that even the lowest dose inoculum of 1 PFU of ZIKV virus resulted in 100% mortality.^47^ Interestingly, in one study, all mice receiving different doses of ZIKV (from 10^4^ to 10^0^ PFU) died at the same time point (100% mortality at day 8),^47^ which is consistent with our current findings. In another study, instead of using immune-deficient transgenic mice, the authors evaluated ZIKV-neutralizing mAbs in wild-type mice treated with an anti-IFN-α-receptor mAb, in which the mice exhibited less severe signs of disease and lower mortality compared to the IFN receptor knockout mice.^25^ A more dramatic difference between antibody groups and the control group would likely be observed in such a model.

## DISCUSSION

The explosive epidemiology of the ZIKV outbreak in South America in 2015–2016 has affected more people than all previously recorded outbreaks combined. ZIKV infection is associated with Guillain–Barré syndrome and microcephaly,^7, 8^ which highlights the threat of ZIKV to global public health. While significant progress has been made, no therapeutics or vaccines are currently available to treat or prevent the disease. Recently, an increasing number of mAbs against emerging viruses, including SARS-CoV, MERS-CoV, and Ebola, have been developed and exhibited high potency *in vitro* and in animal models of infection.^38, 48, 49^ Neutralizing antibodies also play a decisive role in protection from flavivirus infection;^50^ therefore, the development of high-affinity and potently neutralizing antibodies represents a promising strategy to combat ZIKV, for example, to facilitate the design of effective vaccines capable of eliciting a robust and specific antibody response against ZIKV.

Previous studies revealed two classes of broadly neutralizing antibodies to flaviviruses. These broadly neutralizing antibodies target the conserved fusion loop epitope (FLE) in DII or protein E dimer epitope (EDE) in DI/II.^50^ In one study, Barba-Spaeth *et al.*^28^ described a panel of DENV-elicited antibodies targeting FLE or EDE and found that EDE antibodies exhibited more potent cross-neutralization than the FLE antibodies, indicating that EDE antibodies are better suited for developing an epitope-focused vaccine compared to FLE antibodies, which induce poorly neutralizing and strongly infection-enhancing antibodies via antibody-dependent enhancement. However, FLE is the immunodominant epitope in the ZIKV E protein, and most ZIKV-specific antibodies in infected patients were found to be FLE antibodies.^27^ It is therefore arduous to direct specific and potent immune responses to certain epitopes of DI/II without the elicitation of cross-reactive antibody-dependent enhancement antibodies.

Very recently, ZIKV DIII-specific neutralizing mAbs were isolated, and they demonstrated efficient protection in animal models of infection.^25, 26, 27^ Zhao *et al.* reported a panel of mAbs isolated from immunized mice with infectious ZIKV and recombinant DIII. These mAbs specifically bound to three distinct epitopes on DIII and efficiently inhibited the infection of African, Asian, and American ZIKV strains. Furthermore, those mAbs targeting the DIII lateral ridge region conferred complete protection in ZIKV-infected mice.^25^ However, fully human mAbs with minimal immunogenicity in patients would be preferable. Except for isolating neutralizing antibodies from infected or vaccinated subjects, combinatorial antibody libraries and phage/yeast display-based technologies have revolutionized human antibody selection with high binding activity and novel properties.^29^ Herein, through bio-panning of a human Fab library with ZIKV DIII, we successfully selected and characterized a panel of novel germline-like human mAbs with high affinity and specificity against ZIKV DIII. This is the first study to use a phage display screen to identify human antibodies against ZIKV. The four mAbs (m301–m304) recognized three distinct epitopes on DIII and displayed functionally different properties. Since germline mAbs typically exhibit good druggability and low to no immunogenicity, these antibodies, especially the most potent neutralizing mAb, m301, have the potential for further development as antibody-based therapeutics, either alone or in combination with other DIII- or E protein-targeting human mAbs.

The ability of a mAb to bind to a given viral epitope depends on its concentration, the affinity of its interaction with the infectious virus particle, and the accessibility of the epitope on the virion.^51^ While some epitopes are readily accessible on the surface of mature virions, others are partially or completely inaccessible.^25, 42^ However, antibodies that recognize partially or completely occluded sites on the mature virion can still neutralize flavivirus infections because of particle heterogeneity with respect to maturation and/or a dynamic process known as ‘breathing’, which allows for intermittent displays of cryptic epitopes.^52^ Here, our structural studies indicate that the binding epitope of m301 is the C–C′ loop on DIII that is not accessible on the surface of mature virions. Instead, m301 most likely engages the cryptic C–C′ loop epitope in a manner similar to that of the DENV-1-specific neutralizing mAb E111.^42^ Residues on the C–C′ loop are intimately involved in lateral E protein contacts on the mature virion surface. Consequently, their exposure would require substantial reorganization of the particle, which perhaps could occur locally rather than globally.

It is interesting to note that most of the identified antibodies in this study share the same IGHV3–30 gene segment usage. We found that a number of human neutralizing mAbs isolated from flavivirus-infected individuals also used the IGHV3–30 gene segment, for example, mAbs SC4299, SC4311 and SC4353 targeting West Nile DIII,^39^ mAb82.11 targeting DENV DI/II,^40^ and mAb Z3L1 targeting ZIKV DIII.^41^ Notably, the IGHV1-69 and IGHV3-30 genes were frequently used in a number of antiviral antibodies. We previously found that IGHV1-69 allele-specific residues, for example, Phe54 in HCDR2 and Lys73 in FR3, are critical for antigen binding and virus neutralization.^35^ Further structural studies are warranted to explore whether allele-specific residues contribute to the binding of IGHV3-30-encoded antibodies to flavivirus E proteins.

The structural and functional characterization of identified antibodies has implications for the design of effective ZIKV vaccines. E protein DIII was once considered to be the antigen of choice for vaccine development.^53, 54^ However, in a number of studies, it was found that the use of recombinant flavivirus DIII as subunit vaccines only elicited poorly neutralizing antibodies targeting the AB loop of DIII.^19, 55^ These findings suggested that the human antibody repertoire against flaviviruses may actually be directed away from these DIII-neutralizing epitopes, especially an epitope on the lateral ridge region of DIII recognized by many of the strongest flavivirus-neutralizing antibodies.^56^ Therefore, DIII subunit vaccines are not among the leading candidates (and recently approved) for dengue vaccines, nor are they prominent among platforms leading the way for ZIKV vaccine design.^11, 57, 58^ Our results indicate that antibodies targeting the cryptic C–C′ loop are inherently less protective against ZIKV infection *in vitro* and *in vivo* than previously described potent neutralizing antibodies targeting the upper lateral surface of DIII. Although the binding affinities were very high, the neutralizing activities were low. Consistent with low neutralizing activities, these antibodies have only modest protection in mouse models. These results suggest that such antibodies targeting the cryptic epitope on DIII of the ZIKV E protein are probably not suitable as therapeutic agents against ZIKV. As human antibodies targeting the C–C′ loop also exist in ZIKV-infected patients, a current priority is to understand whether such novel antibodies contribute to protective immunity *in vivo*, followed by translating this information into the design of more effective DIII-based vaccine candidates.

In summary, we identified a panel of human mAbs against ZIKV DIII, several of which bind to distinct regions on DIII and have disparate functional activities. By defining a previously unnoticed cryptic C–C′ loop epitope on the E protein DIII, our study has implications for the development of effective ZIKV vaccines.

## Figures and Tables

**Table 1:** Genetic analysis of the heavy and light chain variable regions of ZIKV DIII-specific antibodies

**mAb**	**Variable region**	**Variable region identity (%)**	**D**	**J**	**CDR3**
*V*_*H*_					
m301	HV3-30*03	97.57	D6-6*01	J4*02	AKEVGRSYFDY
m302	HV3-30*04	98.26	D7-27*01	J6*02	AREIGDYYYYGMDV
m303	HV3-64D*06	100.00	D2-2*01	J4*02	VFPSLGYCSSTSCYPPS
m304	HV3-30*03	98.96	D3-10*01	J3*02	ARQRGAFDI
					
*V*_*L*_					
m301	KV4-1*01	97.64		J1*01	QQYYSTPQT
m302	KV3-11*01	94.98		J4*01	QQSYSTPLT
m303	LV2-14*01	98.26		J3*02	SSYTSSSSWV
m304	KV1-12*01	97.85		J3*01	QKYNSAPLT

Abbreviations: complementarity-determining region, CDR; monoclonal antibody, mAb; heavy- and light-chain variable domains, V_H_ and V_L_.
