# Supplementary material for: Neutralization of Zika virus by germline-like human monoclonal antibodies targeting cryptic epitopes on envelope domain III
Source: Emerg Microbes Infect. 2017 Oct 11;6(10):e89–. doi: 10.1038/emi.2017.79 (PMC5658772; doi:10.1038/emi.2017.79)
Supplement: Supplementary Information [file emi201779x1.pdf]

Supplemental Information

Neutralization of Zika virus by germline-like human monoclonal antibodies targeting cryptic epitopes on envelope domain III

Yanling Wu, Shun Li, Lanying Du, Chunyu Wang, Peng Zou, Binbin Hong, Mengjiao Yuan, Xiaonan Ren, Wanbo Tai, Yu Kong, Chen Zhou, Lu Lu, Xiaohui Zhou, Shibo Jiang, Tianlei Ying

FIGURE LEGENDS

Figure S1: Immunogenetic analysis of the heavy and light chain variable regions of m303 and m304 using the IMGT tool.

Figure S2: Immunogenetic analysis of the m301 similar sequences from the IgM repertoire from healthy human neonates and adults. Sequence ID started with CB representing sequences derived from the library of the neonates, and started with HH representing sequences derived from the library of the adults.

(a): Amino acid sequences of HCDR3s that were found similar to that of m301 from the deep sequencing analysis of IgM libraries derived from 33 healthy human adults and 10 neonates, which were analyzed by Clusteral W2.

(b): Germline-rooted circular phylogenetic tree of m301 like antibody sequences found in IgM libraries derived from 33 healthy human adults and 10 newborn babies. The phylogenetic tree was constructed by the Neighbor-Joining method. The numbers labeled on the branch in red representing the probability to gain the topological structure, only the probabilities more than 50% were labeled that means the structures were more reliable.

Figure S3: Immunogenetic analysis of the m302 similar sequences from the IgM repertoire from healthy human neonates and adults.

(a): Amino acid sequences of HCDR3s that were found similar to that of m302 from the deep sequencing analysis of IgM libraries derived from the libraries of the healthy human neonates and adults.

(b): Germline-rooted circular phylogenetic tree of m302 like antibody sequences found in IgM libraries derived from the libraries of the healthy human neonates and adults.

Figure S4: Immunogenetic analysis of the m304 similar sequences from the IgM repertoire from healthy human neonates and adults.

(a): Amino acid sequences of HCDR3s that were found similar to that of m304 from the deep sequencing analysis of IgM libraries derived from the libraries of the healthy human neonates and adults.

(b): Germline-rooted circular phylogenetic tree of m304 like antibody sequences found in IgM libraries derived from the libraries of the healthy human neonates and adults.

Figure S5: Immunogenetic analysis of the m303 similar sequences from the IgM repertoire from healthy human neonates and adults.

(a): Amino acid sequences of HCDR3s that were found similar to that of m303 from the deep sequencing analysis of IgM libraries derived from the libraries of the healthy human neonates and adults.

(b): Germline-rooted circular phylogenetic tree of m303 like antibody sequences found in IgM libraries derived from the libraries of the healthy human neonates and adults.

Figure S6: Alignment of E protein DIII of ZIKV isolated circulating in 2015-2016 ZIKV outbreak.

Figure S1

```
                                 1        10        20        30        40        50         60
                                                                                    a
                                 .........|.........|.........|.........|.........|..........|
m303                             EVQLVESGGGLVQPGGSLRLSCSASGFTFSSYAMHWVRQAPGKGLEYVSAISSNGGSTYYA
Homsap IGHV3-64D*06(100%)        .............................................................
                                 -------------------------------------------------------------
                                 -------------------------------------------------------------

                                61        70        80           90               100       110
                                                      abc                       abcdefg
                                 .........|.........|............|................|.........|
m303                             DSVKGRFTISRDNSKNTLYLQMSSLRAEDTAVYYCVFPSLGYCSSTSCYPPSWGQGTLVTVSS
Homsap IGHV3-64D*06(100%)        ...................................K---------------------------
Homsap IGHD2-2*01                ----------------------------------------.........A-------------
Homsap IGHJ4*02(80.85%)          ------------------------------------------------.FDY...........


                                 1        10        20        30        40        50         60
                                                                                   a
                                 .........|.........|.........|.........|.........|..........|
m304                             EVQLVQSGGGVVQPGRSLRLSCAASGFTFSSYGMHWVRQAPGKGLEWVAVISYDGSNKYYA
Homsap IGHV3-30*03(98.96%)       Q....E .........................A............................
                                 -------------------------------------------------------------
                                 -------------------------------------------------------------

                                 61       70        80           90      100       110
                                                     abc
                                 ........|.........|............|........|.........|
m304                             SVKGRFTISRDNSKNTLYLQMNSLRAEDTAVYYCARQRGAFDIWGQGTMVTVSS
Homsap IGHV3-30*03(98.96%)       ...................................------------------
Homsap IGHD3-10*01               ------------------------------------ITMVRGVII---------
Homsap IGHJ3*02(91.84%)          --------------------------------------D...............
```

Figure S2

```
M301                               AKEVGRSYFDY
CB_M301_sm_521251801014941         AKESGSYYFDY
CB_M301_sm_512162673729430         AKEVRSYYFDY
CB_M301_sm_52206679634125          AKEVGATYFDY
CB_M301_sm_52209324713178          AKEMAGWYFDY
CB_M301_sm_51204744616893          AKEHSGSYFDY
CB_M301_sm_512021468110124         AKESGGSYFDY
CB_M301_sm_512091228642337         AKEVPLGYFDY
HH_M301_sm_121012706238222         AKSIVEGYFDY
HH_M301_sm_12214977046451          AKDANGGCFDY
CB_M301_sm_51107272728024          AKETEGGYFDY
CB_M301_sm_521193122337273         AKERNGDYFDY
CB_M301_sm_512211981614555         AKEGLGYYFDY
CB_M301_sm_512042919322467         AKELLGAYFDY
CB_M301_sm_522261242822995         AKERLQYYFDY
CB_M301_sm_511202546913236         AKEAEDYYFDY
CB_M301_sm_51123417813341          AKEAHGYYFDY
CB_M301_sm_511161095729483         AKEGQGWYFDY
CB_M301_sm_512262328726090         AKEGQLVYFDY
CB_M301_sm_511172327722344         AKEGNWHYFDY
HH_M301_sm_322252190720709         AKVRDSSLFDY
HH_M301_sm_311092372325931         AKESPTVAFDY
HH_M301_sm_321211820330081         AKDSRRWSFDY
HH_M301_sm_12105493910053          AKDLDNWEFDY
HH_M301_sm_32227325438679          AKDRNWLGFDY
HH_M301_sm_111112631117368         AKDRRTTVFDY
HH_M301_sm_311123002535972         AKGSSWYPFDY
HH_M301_sm_322121011512269         AKDSYDYPFDY
HH_M301_sm_121122956938662         AKSKQAIPFDY
HH_M301_sm_3122699736994           AKNGAYNDFDY
HH_M301_sm_32214300259702          AKVGVVNHFDY
HH_M301_sm_11109244853231          AKDGHSNTFDY
HH_M301_sm_121123142613781         AKRGYSYGFDY
                                   **      ***
```

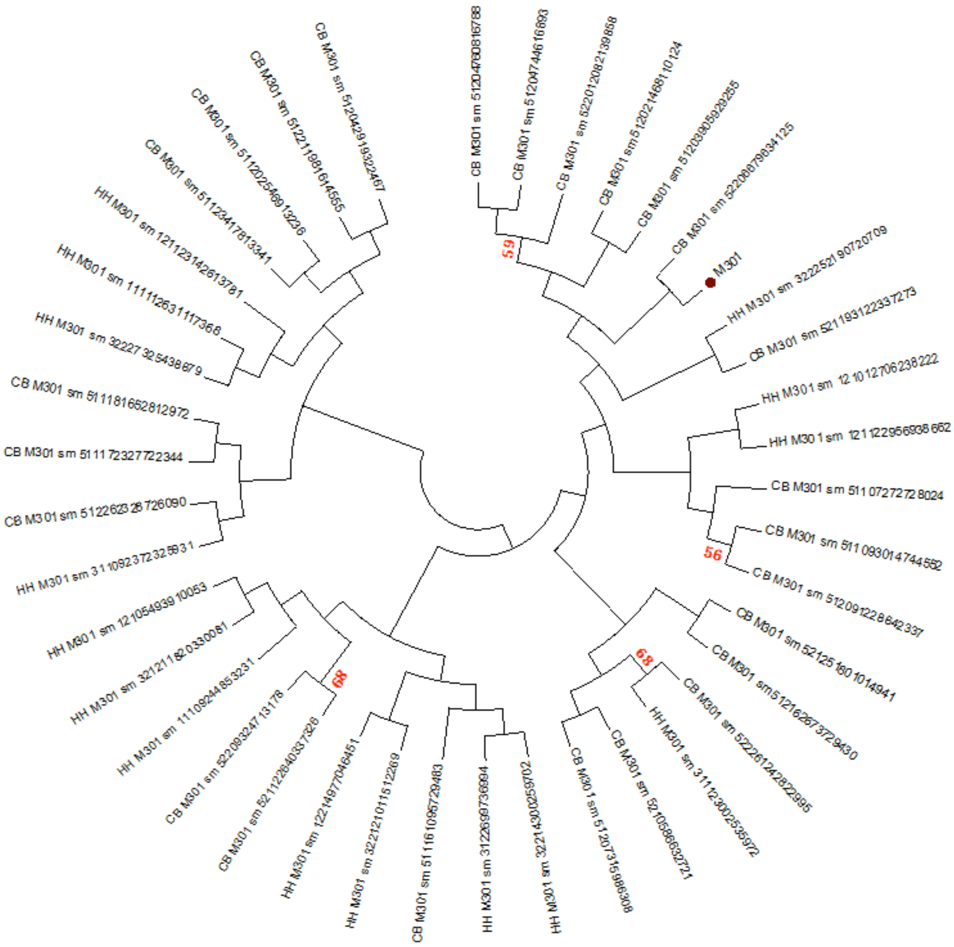

Figure S3

```
M302                              AREIGDYYYYGMDV
CB_M302_sm_511062610848245        ARENGVYYYYGMDV
HH_M302_sm_12124250321447         AREWGYYYYYGMDV
HH_M302_sm_311011389017368        AREHGHYYYYGMDV
HH_M302_sm_121151203314607        ARESGSYYYYGMDV
CB_M302_sm_52101686729466         ARELGDFYYYGMDV
HH_M302_sm_122172396738205        AREAPFYYYYGMDV
HH_M302_sm_11209345721342         AREAYYYYYYGMDV
HH_M302_sm_122163110130943        AREAAHYYYYGMDV
HH_M302_sm_311192840228797        ARESAHYYYYGMDV
HH_M302_sm_112191441819056        AREAASYYYYGMDV
HH_M302_sm_1221222508664          AREPDTYYYYGMDV
HH_M302_sm_111212038517579        AREPDAYYYYGMDV
HH_M302_sm_31220181722914         AREPMGYYYYGMDV
HH_M302_sm_312011817232701        ARELSPYYYYGMDV
HH_M302_sm_32221465513359         ARECIPYYYYGMDV
HH_M302_sm_122251814225369        ARERRAHYYYGMDV
HH_M302_sm_312022250633176        ARERGAYYYYGMDV
HH_M302_sm_312122499245467        AREKGGSYYYGMDV
HH_M302_sm_31114984122344         AREEDGDYYYGMDV
HH_M302_sm_312271226642372        AREGDGAYYYGMDV
HH_M302_sm_122052136943304        AREVWFAYYYGMDV
HH_M302_sm_3222828996941          ARENNRSYYYGMDV
HH_M302_sm_12105530440543         AREELRFYYYGMDV
HH_M302_sm_112231057142917        ARELTRPYYYGMDV
HH_M302_sm_322031089628569        ARELLSPYYYGMDV
HH_M302_sm_311272458618423        AREGTHDYYYGMDV
HH_M302_sm_121182204917087        AREGLHDYYYGMDV
HH_M302_sm_112271073416753        ARELRLKYYYGMDV
HH_M302_sm_12112569031787         AREGQLVYYYGMDV
HH_M302_sm_12218191471789         AREGRSYYYYGMDV
HH_M302_sm_311272437339225        AREGSSSYYYGMDV
CB_M302_sm_512201458038363        AREGDSTYYYGMDV
CB_M302_sm_521233008622678        AREGGSTVLGGMDV
                                  ***       ****
```

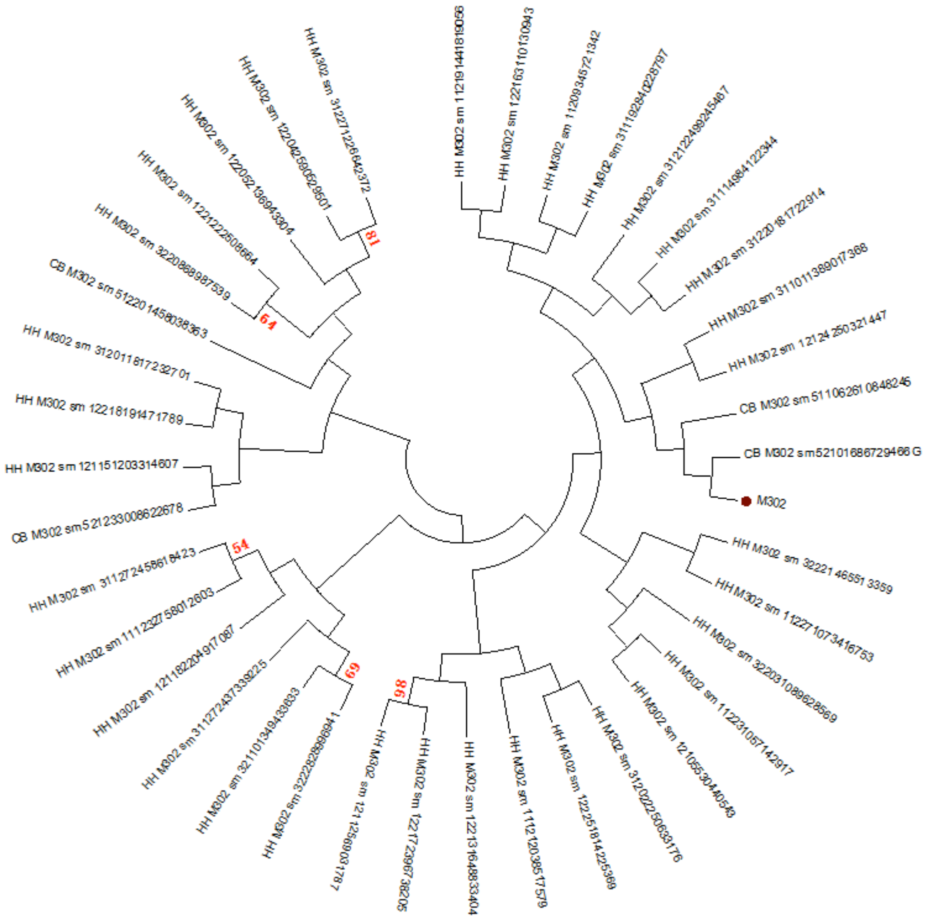

Figure S4

```
M304                              ARQRGAFDI
CB_M304_sm_51221291324842         ARQRGAFDI
CB_M304_sm_52122250734177         ARERGAFDI
HH_M304_sm_31205139203655         ARERGAFDI
CB_M304_sm_51205130981578         ARSRGAFDI
HH_M304_sm_12124231042067         ARSRGAFDI
CB_M304_sm_52120171377627         ARKRGAFDI
HH_M304_sm_11110165082971         ARDRGAFDI
CB_M304_sm_51203252461211         ARDRGAFDI
HH_M304_sm_12105202938066         ARTRGAFDI
HH_M304_sm_1110282983108          ARVRGAFDI
HH_M304_sm_12219245054275         ARNRGAFDI
HH_M304_sm_32117323902132         ARIRGAFDI
CB_M304_sm_51226934342794         ARARGAFDI
HH_M304_sm_12128983119478         ARARGAFDI
HH_M304_sm_11114225162201         ARGRGAFDI
CB_M304_sm_51224136973532         ARGRGAFDI
                                  ** ******
```

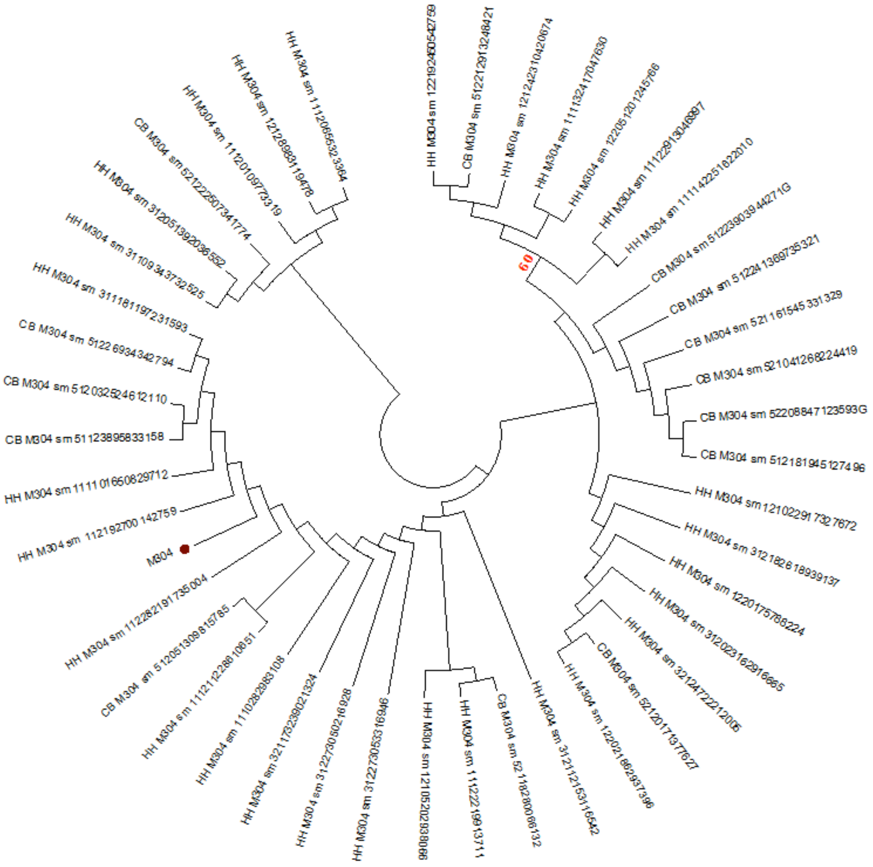

Figure S5

```
m303                             VFPSLG--YCSSTSCYP-PS--
CB_M303_sm_522183009721113       --AKYHR-YCSSTSCP--EFDY
CB_M303_sm_51224273674848        --AKELG-YCSSTSCY--AFDY
CB_M303_sm_521042613915803       --ARAG--YCSSTSCFT-PFDY
CB_M303_sm_52216293766572        --ARRG--YCSSTSCYW-DFDY
CB_M303_sm_51203577126582        --ARVGGGYCSSTSCY---FDY
HH_M303_sm_111101884226723       --ARVG---CSSTSCYAAGFDY
HH_M303_sm_11115103285429        --ARGD---CSSTSCYVGEFDY
                                   .      ******
```

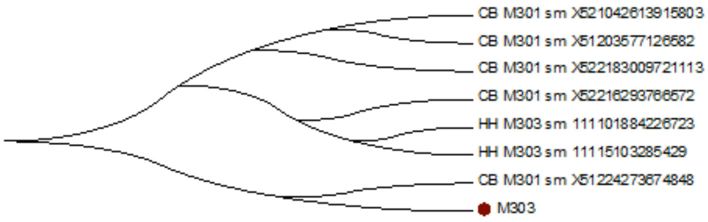

*CB: data from newborn babies

HH: data from healthy adult donors

Figure S6

```
               303   310       320       330        340      350       360
               .........|.........|.........|.........|.........|.........|
SZ01           VSYSLCTAAFTFTKIPAETLHGTVTVEVQYAGTDGPCKVPAQMAVDMQTLTPVGRLITAN
FLR            ---------------------------V--------------------------------
PAN2015        ---------------------------V--------------------------------
R103451        ---------------------------V--------------------------------
PRVABC59       ---------------------------L--------------------------------

                     370       380       390       400
               .........|.........|.........|.........|...
SZ01           PVITESTENSKMMLELDPPFGDSYIVIGVGEKKITHHWHRSG
FLR            ------------------------------------------
PAN2015        ------------------------------------------
R103451        ------------------------------------------
PRVABC59       ------------------------------------------
```
